# The Book World of Medicine and Science

**Published:** 1898-02-19

**Authors:** 


### THE BOOK WORLD OF MEDICINE AND SCIENCE.

TWENTIETH CENTURY PRACTICE OF MEDICINE. Edited by THOMAS L. STEDMAN, M.D. Vol. IX. Diseases of the Digestive Organs. (London: 1897. Sampson Low, Marston, and Co.) Pp. 820, with 57 illustrations.

This massive volume amply maintains the reputation of the series to which it belongs. The title, chosen no doubt for the sake of brevity, is a little misleading, and it will be as well to state that the subjects treated comprise the local diseases of the mouth, diseases of the intestines, hernia, diseases of the spleen, liver, and gall bladder, and movable kidney. It will thus be seen that there is a considerable admixture of surgical matter, and, in fact, about one-third of the book is written by surgeons. The chapter on the mouth is by no less eminent an authority than Professor Mikulicz, of Breslau, certainly one of the most brilliant of living Continental surgeons, who writes in collaboration with Dr. Kümmel of the same University. It need hardly be said that the article is fully abreast of modern surgical requirements, though the scope of the book renders it encyclopædic rather than detailed. The diseases of the intestines are by Professor Ewald, of Berlin, another name which guarantees accuracy of scholarship and experienced judgment. It also, however, suffers from compression, being limited to less than 200 pages, of which 40 are given to appendicitis. This is again the result of the entire system being a somewhat extended encyclopædia rather than a series of monographs. One cannot but contrast Professor Ewald's excellent sketch with the masterly and complete account of the same subject by Nothnagel in the latter's recently published Handbuch. It is obviously very difficult to give a complete statement of the modern aspect of appendicitis and allied conditions within the limits here assigned. The next article, that on "Hernia," by Drs. Gibney and Walker, of New York, is an excellent summary admirably illustrated; among other noteworthy features it contains the best description of Bassini's operation which we have seen in English. Dr. Stengel's account of the diseases of the spleen calls for no comment; but the chapter on diseases of the liver by Professor Semmola and Dr. Gioffredi is the most important in the book. It occupies nearly 340 pages, of which 135 are devoted to a general consideration from the point of view of anatomy, physiology, pathology, symptomatology, and therapeutics. About a fifth of this section is devoted to the consideration of jaundice, and the authors lay it down with all the weight of their authority that there is no such thing as hæmatogenous icterus, but that as "biligenesis"—a mongrel word which it is to be hoped will not survive—is the normal function of the liver, so jaundice is the result of interference with it. The treatment of hepatic disease by milk diet, which Professor Semmola has so long and ardently advocated, is, of course, recommended here and supported by rational scientific argument. It may be mentioned that this chapter bears evidence of a closer study of English medical literature than is usual with foreign writers. We must, however, express our regret that the authors should have so far yielded to the modern craze for classification as to enumerate twelve varieties of cirrhosis of the liver. The succeeding chapter treats of diseases of the gall-bladder, and is from the pen of Professor Murphy, of Chicago, who writes succinctly but admirably; an illustration is given of his new cholecystoscope. The last article, that on movable kidney, is by Mr. Kendal Franks, who again brings forward his well-known views as to its mechanical production; he also gives useful directions for treatment. On the whole, the book, as has already been said, fully sustains the value of the system as a work of reference.

NERVOUS AFFECTIONS OF THE HAND AND OTHER CLINICAL STUDIES. By GEORGE VIVIAN POORE, M.D., F.R.C.P., Physician to University College Hospital, &c. (London: 1897. Smith, Elder, and Co.) Pp. 308, with 9 illustrations. Price 7s. 6d.

Dr. Poore's writings on purely medical subjects are so infrequent that this volume comes as a welcome reminder of his exceptional clinical insight and his refreshing breadth of view. The book consists entirely of articles which had previously seen the light of publication in various medical journals, but which certainly deserved a better fate than to be buried among a mass of ephemeral literature. Among them the lectures occupying the first 128 pages are conspicuous, embodying as they do the most important studies which Dr. Poore has hitherto contributed to the progress of medicine, studies with which his name will always be associated. We refer to the series of six lectures on occupation neuroses, appropriately headed by his well-known Bradshawe lecture, from which the title of the book is taken. Dr. Poore was one of the first to study writers' cramp from the clinical point of view. Up to 1887 he had seen no fewer than 168 cases of this affection, including a few in which the writing difficulty was due to other than local causes. The second of the lectures here reprinted (on "Professional Neuroses") comprises by far the most scientific analysis of the disease which has hitherto appeared, in England at any rate. Other addresses add largely to our knowledge of the cramps affecting hammermen, goldbeaters, tailors, compositors, and piano players; of the last rare affection Dr. Poore had had in 1886 experience of 21 cases. And one feels throughout that this comparatively vast material has been worthily used, every case being subjected to that critical and impartial analysis on which alone a rational treatment can be based. The remaining lectures in the volume touch upon very varied themes. Five of them refer to different forms of intoxication ranging from phosphorus to uric acid, and of these the most interesting relates the case of a young athlete who, after running 10 miles in a comparatively untrained condition, became suddenly comatose, and to some extent convulsed. He remained in a condition of cerebral irritation for twenty-four hours, eventually recovering during the passage of a stomach tube. Nothing abnormal was found on examination, and Dr. Poore skilfully and convincingly argues that this was a case of auto-intoxication, the patient being poisoned by the products of his muscular exertions. These were not removed as usual, owing to his lack of "condition," it being noticed that during the exercise he scarcely perspired at all. The case affords an excellent example of the author's clinical method. It finds its therapeutic counterpart in two lectures on the treatment of heart disease and of phthisis, which attracted considerable attention when they were delivered, and are well worthy of close study. In the treatment of what may be called "the hospital heart," Dr. Poore eulogises rest above all other remedies, and his careful study of a case treated without drugs lends much support to his view that with a large proportion of patients cardiac tonics are by no means indispensable. As regards pulmonary tuberculosis, he pays, of course, much attention to prophylaxis; he also records many striking instances of improvement under the use of garlic as a bactericide. We are tempted to quote more of Dr. Poore's views on this and other topics, but we hope that enough has been said to indicate that he has written a book which is as original in its ideas as it is vigorous and lucid in its diction, and in which an enormous amount of scientific knowledge is turned to practical clinical use.

